# Verses

**Published:** 1906-03

**Authors:** 


### VERSES.

### Sowing and Reaping.

"Sow a thought and reap an act,
Sow an act and reap a habit,
Sow a habit and reap a character,
Sow a character and reap a destiny."

*Exchange.*

---

### Jealous Men.

"I know some men who lay in wait
And get up soon and stay up late
To catch some fellow they can hate
For going with a faster gait."

*Exchange.*

---

### Alas.

My dentist bill no more than paid,
Than that huge molar plugged with gold
Is up and making horrid raid
On all the nerves my mouth will hold.
Ah, woe is me! With aches grown wild,
Comes home to me this ancient truth:
How sharper than a serpent's child
It is to have a thankless tooth!

*American Dental Journal.*

### His Wife.

He calls her "Little Sunshine,"
Not because her flashing eyes
In the splendor of their brightness
Bear the light of sunny skies.

He calls her "Little Sunshine,"
Not because her golden hair
Has the glory of the sunlight
In its masses painted there.

He calls her "Little Sunshine,"
From no likeness to the glim,
Save that now and then she makes it
Just a bit too warm for him!

*J. W. Foley.*

---

### A Goat-Sheep.

Onc't I hed er Billy-goat,
Long ha'r'd an' long horned,
He stepped eround de yaard an' lot
Lak he wus boss sence he wus borned.

He butt de cow an' run de sheep,
Side-step de mule an' ram de shoat,
Dere warn't er fence he wouldn't leap,
He wus er mos' outrageous goat.

One day he jess hammered me,
Es I stooped ter pick up cawn,
De moons an' stars I did see,
Wus de sparkin'ess I ebber sawn.

Well, I jess cotch dat Mr. Billy,
An' I doped him in er heap,
An' saw'd dem horns wile he was silly
An' turn im ter er sad-eyed sheep.

*B. H. Teague.*

